# Electrodermal Activity as a Metric of Arousal in Autistic Children Prior to Dental Treatment

**DOI:** 10.3390/s26196172

**Published:** 2026-09-29

**Authors:** Elizabeth B. Isralowitz, Sharon A. Cermak, John Sideris, Grace T. Baranek, José C. Polido, Jianina Marie Y. Ferrer, Leah I. Stein Duker

**Affiliations:** 1 Mrs. T.H. Chan Division of Occupational Science and Occupational Therapy, University of Southern California, 1540 Alcazar St., CHP-133, Los Angeles, CA 90089, USA; cermak@chan.usc.edu (S.A.C.); sideris@chan.usc.edu (J.S.); baranek@chan.usc.edu (G.T.B.); nferrer@alumni.usc.edu (J.M.Y.F.); lstein@chan.usc.edu (L.I.S.D.); 2 Division of Dentistry, Children’s Hospital Los Angeles, 4650 Sunset Blvd, MS#116, Los Angeles, CA 90027, USA; jpolido@chla.usc.edu

**Keywords:** electrodermal activity, autism, arousal, anxiety, language, behavioral symptoms, sensory processing, galvanic skin response, sensors

## Abstract

**Highlights:**

This study aimed to examine autistic children’s arousal characteristics during the initial rest period before routine dental treatment and their relation to behavior.

**What are the main findings?**

- Increased autism traits predicted tonic EDA level (skin conductance level; SCL) trajectories over time: children with fewer autism traits showed increasing SCL across the rest period, whereas children with higher autism traits displayed physiological habituation (declining SCL). Additionally, generalized anxiety significantly predicted lower phasic EDA activity (non-specific skin conductance response frequency; NS-SCR). After controlling for cognitive functioning (IQ), a significant quadratic (inverted U-shaped) relationship between anxiety and NS-SCR frequency emerged.
- No significant relationships were observed between EDA measures and sensory over-responsiveness or expressive language.

**What are the implications of the main findings?**

- Baseline physiological arousal in autistic children appears to reflect distinct within-population profiles and trajectory patterns linked to autism traits and anxiety, rather than a uniform hyper- or hypo-arousal state. In addition, individual differences in cognitive functioning (IQ) may obscure non-linear relationships between physiological arousal and anxiety and should be considered in future EDA studies.
- Methodological and contextual factors (e.g., measurement approach, environmental conditions, participant heterogeneity) likely influence EDA outcomes, underscoring the need for standardized protocols and multi-measure approaches in future research.

**Abstract:**

Several prominent theories have asserted atypical arousal as a biological feature of autism. However, there is mixed evidence regarding atypical arousal patterns, particularly resting-state arousal, in autistic children, with research increasingly using psychophysiological measures, such as electrodermal activity (EDA), to help elucidate arousal levels in individuals who might not otherwise be able to identify such states. This study analyzed extant EDA data from 135 autistic children (6–12 years) collected during a baseline rest period in a dental environment to determine the relationships between tonic (mean skin conductance level; SCL) and phasic (frequency of non-specific skin conductance responses; NS-SCRs) arousal and participant characteristics, including sensory over-responsiveness, autism traits, expressive language, and general anxiety. EDA data were collected using pre-gelled electrodes placed on the distal phalanges of the participant’s non-dominant hand using the BIOPAC MP150 system. Data preprocessing and epoch analyses were conducted using BIOPAC AcqKnowledge software. Conditional linear and, for the outcome variable anxiety, quadratic growth models were used to assess the temporal (within-participant across observations) relationship between EDA and behavioral variables. The results revealed a significant time-by-autism trait interaction for SCL, as well as linear and quadratic relationships between anxiety and NS-SCRs. No significant relationships were found between EDA arousal parameters and sensory over-responsiveness or expressive language abilities. A significant covariance relationship between SCL and NS-SCR frequency was also observed. Collectively, these results support previous characterizations of unique, within-autism-population arousal profiles related to autism traits, anxiety, and tonic-to-phasic covariance patterns.

## 1. Introduction

Autism spectrum disorder is a heterogeneous neurodevelopmental condition in which individuals experience significant differences in social communication and restricted and repetitive behaviors (RRBs) [1,2]. Variations in resting-state arousal profiles may influence adaptive, social communication, and cognitive skills [2,3]. A better understanding of autism-related participant characteristics associated with atypical arousal can help inform our understanding of autistic heterogeneity and arousal-related co-occurring conditions.

### 1.1. Electrodermal Activity as a Measure of Sympathetic Arousal

Electrodermal activity (EDA) is one of the most widely used psychophysiological measures of sympathetic nervous system activity. EDA refers to measurements of autonomic changes in the skin’s ability to conduct electrical current and indicates sympathetic nervous system (SNS) arousal. Arousal refers to an individual’s attentiveness towards internal and external stimuli [4]. Sympathetic arousal mechanisms contribute to emotional and attentional regulation, which are critical for successful adaptive functioning and social interactions [4,5].

EDA signals are commonly divided into two components: tonic and phasic activity. The tonic EDA level is quantified using skin conductance level (SCL), which represents the slow-changing background level of the skin’s electrical conductivity. SCL provides a continuously moving baseline measurement that reflects general changes in autonomic arousal [6,7]. Phasic activity (e.g., skin conductance responses [SCRs]) consists of observable changes in amplitude that occur in response to environmental or internal events or stimuli. Non-specific skin conductance responses (NS-SCRs) occur without identifiable eliciting stimuli and are frequently assessed in more naturalistic research [8,9]. Both SCL [10,11] and SCR-related measures are considered markers of SNS arousal [5,12]. Regulation of dynamic and flexible adaptive responses to one’s environment relies on an optimal state of arousal, which is controlled, in part, by interactions between the central and peripheral neural systems [2].

### 1.2. Sympathetic Arousal, Autism, and Related Characteristics

Several autism-related participant characteristics (e.g., social interactions, communication, sensory responsivity, anxiety, RRBs) have been linked to autonomic arousal in autism and were examined in the present study. Prior research examining these relationships has produced mixed findings. For example, research suggests that anxiety may be characterized by a non-linear, inverted U-shaped relationship with physiological arousal, in which arousal increases to an optimal point and then diminishes at higher levels, potentially reflecting adaptive disengagement and reduced effortful control [13,14,15,16]. Findings regarding sensory responsivity have been inconsistent, with studies reporting patterns of hyper-arousal [17], hypo-arousal [11,18], little within-population variation [16], and distinct high- and low-arousal responder subgroups [12,19,20,21], as well as mixed associations between EDA and sensory responsivity [11,17,22]. Emerging evidence also suggests that atypical autonomic responses may be related to expressive language abilities, with lower arousal associated with poorer language and pragmatic functioning [23,24,25] and communication-related autism traits (e.g., altered social communication) [10,26,27]. Finally, studies examining autistic traits have yielded mixed results, although several have found that greater EDA reactivity is associated with increased autistic traits and higher levels of RRBs [10,22,24,26,28].

### 1.3. Resting-State Tonic and Phasic EDA Arousal

Although tonic (SCL) and phasic (SCR) components of EDA are derived from the same physiological signal [9,29,30,31], evidence suggests that they reflect distinct neurophysiological processes and may exhibit different patterns of association with various autism-related participant characteristics [28,32,33]. For example, studies of autistic children have identified distinct tonic and phasic response profiles [12,19,20,21]: higher resting arousal is associated with greater reactivity, faster response latencies, and slower habituation, whereas lower resting arousal is associated with reduced reactivity, slower latencies, and faster habituation [12,19,20].

Studies of resting-state EDA in autistic children have primarily focused on comparisons with non-autistic groups and have yielded inconsistent findings, with some studies observing no relationship and others finding evidence of both hypo-arousal and hyper-arousal patterns [2]. Few studies have directly examined associations between resting-state arousal and autism-related characteristics (e.g., sensory responsivity, communication, anxiety), with limited evidence linking baseline SCL to sensory responsivity [2,17]. Although these inconsistencies may partly reflect methodological differences across studies, they also underscore the heterogeneity of autonomic arousal patterns within the autistic population [2].

### 1.4. Rationale

Despite several theories proposing atypical autonomic functioning in autism [34,35,36], recent reviews conclude that there is insufficient evidence to support generalized hyper-arousal or hypo-arousal models [2,37]. In their review of the resting-state literature, Arora and colleagues [2] suggest that individual differences in resting-state arousal may contribute to heterogeneity in the autistic phenotype and influence the development of adaptive, social, and cognitive skills. Accordingly, a better understanding of the relationships among resting-state arousal, autistic traits, and related participant characteristics (e.g., sensory responsivity, expressive language, anxiety) may have important implications for autism identification and intervention. The present study extends previous work by examining resting-state arousal and its behavioral correlates in a large sample (*n* = 135) of school-aged autistic children during a baseline rest period in a dental setting. Based on the existing literature, the current study proposes the following hypotheses.

1. Tonic EDA level and phasic activity:
  - Participants with higher phasic activity (NS-SCR frequency) will show higher tonic EDA level (SCL) [9,14].
2. Tonic EDA level and sensory over-responsiveness, autism traits, expressive language, and anxiety:
  - Participants with higher sensory over-responsiveness scores (e.g., [17]) and autism traits [26] will demonstrate higher tonic EDA levels;
  - As anxiety scores increase, mean SCL will follow to a fulcrum, after which participants with increasingly high anxiety scores are predicted to have decreasing tonic EDA levels. Therefore, following a quadratic inverted ‘U’ shape, participants at both the lower and higher ends of the anxiety score continuum will display lower SCL [13,14].
3. Phasic EDA activity and sensory over-responsiveness, autism traits, expressive language, and anxiety:
  - Participants with higher sensory over-responsiveness scores (e.g., [27]) and autism traits [26] will demonstrate higher phasic activity (i.e., more frequent NS-SCRs);
  - As anxiety scores increase, NS-SCR frequency is predicted to follow an inverted ‘U’ shape curve, peaking at moderate anxiety before decreasing. Consequently, participants at both the lower and higher extremes of the anxiety continuum will display fewer NS-SCRs [13].

## 2. Materials and Methods

This study used extant behavioral (i.e., autism traits, generalized anxiety, sensory over-responsiveness, expressive language) and baseline resting-state EDA (SCL and NS-SCR) data from a randomized crossover control trial examining the effectiveness of a sensory-adapted dental environment (SADE) on physiological and behavioral distress during dental visits with autistic children [38].

### 2.1. Participants

The study was conducted at the dental clinic of a large urban hospital. Participants were recruited from the on-site dental clinic, community settings, and other clinics. Children aged 6 to 12 years with a research-confirmed autism diagnosis, at least one prior dental cleaning, and whose family spoke English or Spanish were considered for study inclusion. Children using anticholinergic medications, with or scheduled for orthodontic braces, with an enrolled sibling, significant motor impairments, complicated oral conditions, a medical condition placing the child at an increased risk in the study (e.g., uncontrolled seizures), or an identified genetic (non-autism-related), endocrine, or metabolic disorder were excluded. Of the 220 recruited participants, 162 completed the study, and 135 had usable EDA data from the control condition (regular dental environment) baseline period used in this analysis. The demographic information for participants is shown in Table 1.

### 2.2. Procedures

EDA data were collected at a 100 Hz sampling rate using pre-gelled electrodes on the distal phalanges of the participant’s index and middle fingers of their non-dominant hand using the BIOPAC MP150 system (BIOPAC Systems, Inc., Goleta, CA, USA). Data were preprocessed in the BIOPAC AcqKnowledge software version 5.0 (BIOPAC Systems, Inc., Goleta, CA, USA)using a low-pass finite impulse response (FIR) filter. Following preprocessing, analyses were performed offline using the BIOPAC AcqKnowledge software to derive SCL, NS-SCR frequency (≥0.05 μS amplitude), and dental cleaning phase time duration (e.g., baseline); recordings were then visually inspected to confirm accurate detection of NS-SCRs.

Based on the SADE-2 study protocol, EDA data collection began prior to the documented and analyzed pre-rest/baseline period with a 5–10 min acclimation period for all participants. Using 60 s epochs, we analyzed the average baseline SCL and NS-SCR frequency collected during a three-minute baseline rest period immediately preceding dental care.

#### Measures

Predictor variables included caregiver-reported Sensory Over-Responsivity Scales (SensOR; [39]), expressive language (Vineland Adaptive Behavior Scales, 2nd ed.–Expressive Language; VABS-II [40]), generalized anxiety (Child and Adolescent Symptom Inventory-4: Autism Anxiety Scale; CASI-Anx [41]), and observed autism traits (Autism Diagnostic Observation Scale, 2nd ed.; ADOS-2; Calibrated Severity Score (CSS) [42]), including social interactions, communication, play skills, and RRBs. Supplemental Table S1 displays measure descriptions.

Other participant measures included IQ (Wechsler Abbreviated Scale of Intelligence, 2nd ed.; WASI-II [43]) and caregiver-reported dental anxiety (Children’s Fear Survey Schedule–Dental Subscale; CFSS-DS [44]).

### 2.3. Data Analysis

As is best practice, model diagnostics were performed to ensure the data met the assumptions necessary for running the longitudinal growth models. This included confirming the normality of Level 1 residuals and Level 2 random effects using a normal probability plot and plotting the standardized residuals against predicted values to assess homoscedasticity and verify the adequacy of the variance–covariance structure. Descriptive statistics were run for caregiver-reported demographic information, primary predictor and outcome variables, and considered covariates. To demonstrate the range of individual responses across the various EDA arousal parameters, straw plots were also generated.

To statistically account for the temporal nature of sudomotor nerve responses within each participant across observations/epochs, a series of conditional growth models were fit to the data to model the relationship between (1) EDA tonic (SCL) and phasic (NS-SCR) arousal, (2) tonic EDA level and each behavioral measure (i.e., sensory over-responsiveness, autistic traits, expressive language), and (3) phasic activity and each behavioral measure. A multilevel framework for change was used for growth modeling, with Level 1 (within-person level) estimating individual EDA arousal trajectories across the 3 min baseline and Level 2 (between-person level) conditioning the individual change parameters on individual differences in the predictor variables.

For descriptive and visual representation, following the ADOS-2 manual, autism severity scores were categorized into three groups: low (3–4), moderate (5–7), and high (8–10) severity [42]. CASI-Anx scores were split into mathematical tertiles to visually represent the relationship between anxiety and NS-SCR frequency.

Based on previous anxiety-related EDA activation research in autistic children [13,14], a quadratic term was included in our anxiety growth model analyses. To assess the possibility of variation in arousal due to dental fear-related reactions (CFSS-DS scores) and IQ (WASI-4 scores), these variables were considered as covariates. Finally, to ensure our analyses were not unduly affected by outliers, we conducted a post hoc analysis of median SCL for any non-significant mean SCL results.

## 3. Results

### 3.1. Participant Characteristics

Descriptive statistics for each measure are shown in Table 2.

### 3.2. Tonic EDA Level and Phasic Activity Results

A significant temporal relationship was observed between tonic (SCL) and phasic (NS-SCR) arousal. There was a significant conditional main effect of NS-SCR frequency on mean SCL (*b* = 0.11, *p* < 0.01), as well as an interaction effect for time × NS-SCR (*b* = 0.001, *SE* = 0.00, *t*(167.06) = 3.71, *p* < 0.01). Additionally, the main effect of time was negative (*b* = −0.005, *p* = 0.01), as shown in Table 3.

### 3.3. Tonic EDA Level and Sensory Over-Responsiveness, Autism Traits, Expressive Language, and Anxiety

**Figure 1:**
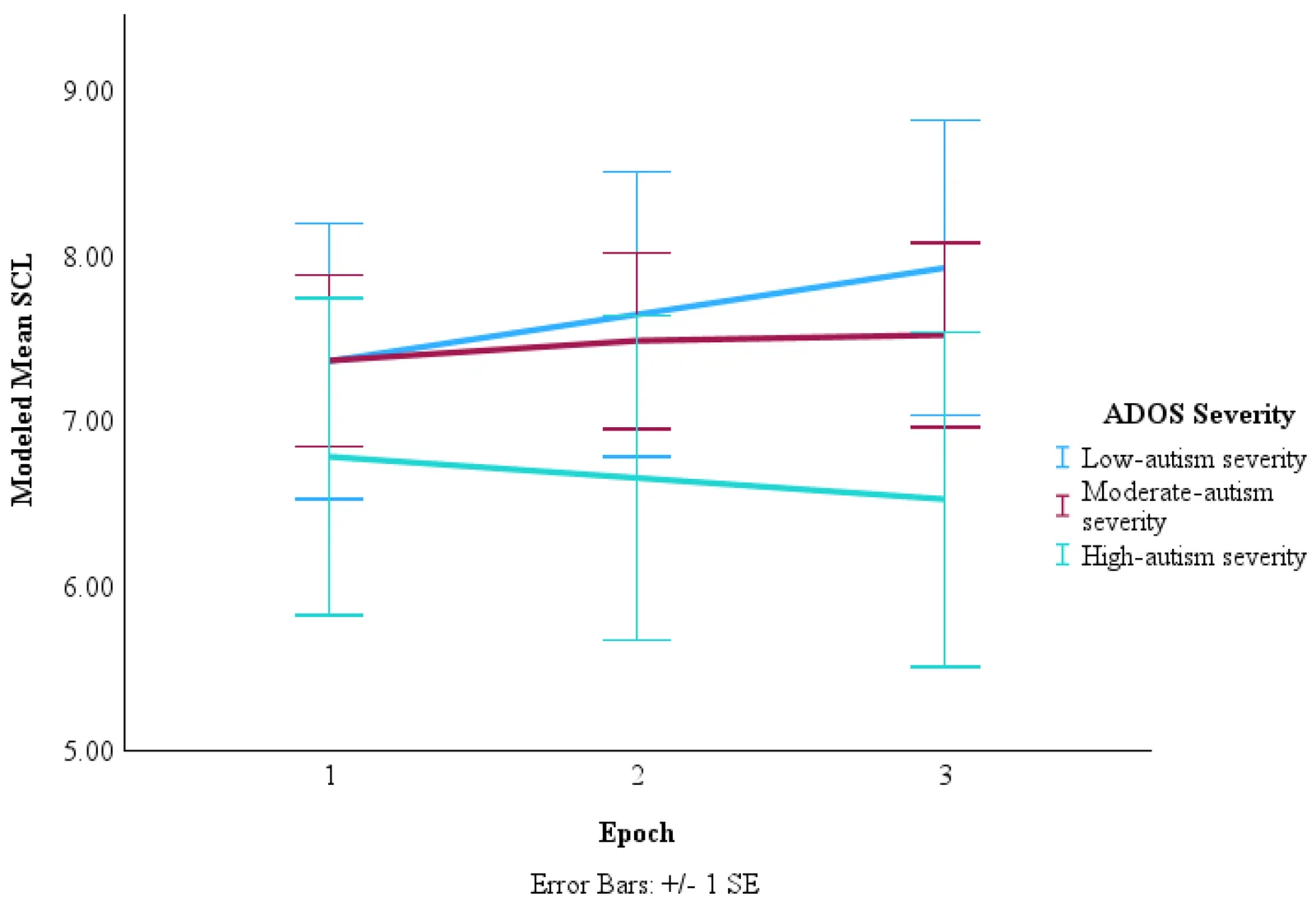
Graphical representation of changes in modeled skin conductance level (SCL) by autism trait severity group (Autism Diagnostic Observation Scale, ADOS) across the three epochs.

A significant interaction effect was observed for time × autism traits predicting mean SCL (*b* = −0.001, *SE* = 0.001, *t*(130.42) = −2.12, *p* = 0.04). While the conditional main effect of time was positive (*b* = 0.01, *p* = 0.02), the negative interaction term demonstrates that increased autism traits were associated with a less positive slope (i.e., a flatter or more downward trajectory) in mean SCL over time, as shown in Table 4 and Figure 1.

There were no significant main effects or time interactions for tonic EDA level (mean or median SCL) and sensory over-responsiveness (mean SCL: *b* = −0.01, *p* = 0.84; median SCL: *b* = −0.01, *p* = 0.85) or expressive language (mean SCL: *b* = 0.03, *p* = 0.82; median SCL: *b* = 0.04, *p* = 0.77), as shown in Table 4. There were also no significant main or interaction effects for the linear or quadratic growth models for mean SCL and anxiety scores (linear: *b* = 0.00, *p* = 0.99; quadratic: *b* = 0.21, *b*^2^ = −0.004, *p* = 0.10) or median SCL and anxiety scores (linear: *b* = 0.003, *p* = 0.94; quadratic: *b* = 0.23, *b*^2^ = −0.005, *p* = 0.09), as shown in Table 5. Inclusion of CFSS-DS and WASI-4 scores as covariates did not significantly improve model fit or significance testing for the predictor variables, which were all pseudo R^2^ ≤ 0.05, *p* ≥ 0.07.

### 3.4. Phasic EDA Activity and Sensory Over-Responsiveness, Autistic Traits, and Expressive Language

**Figure 2:**
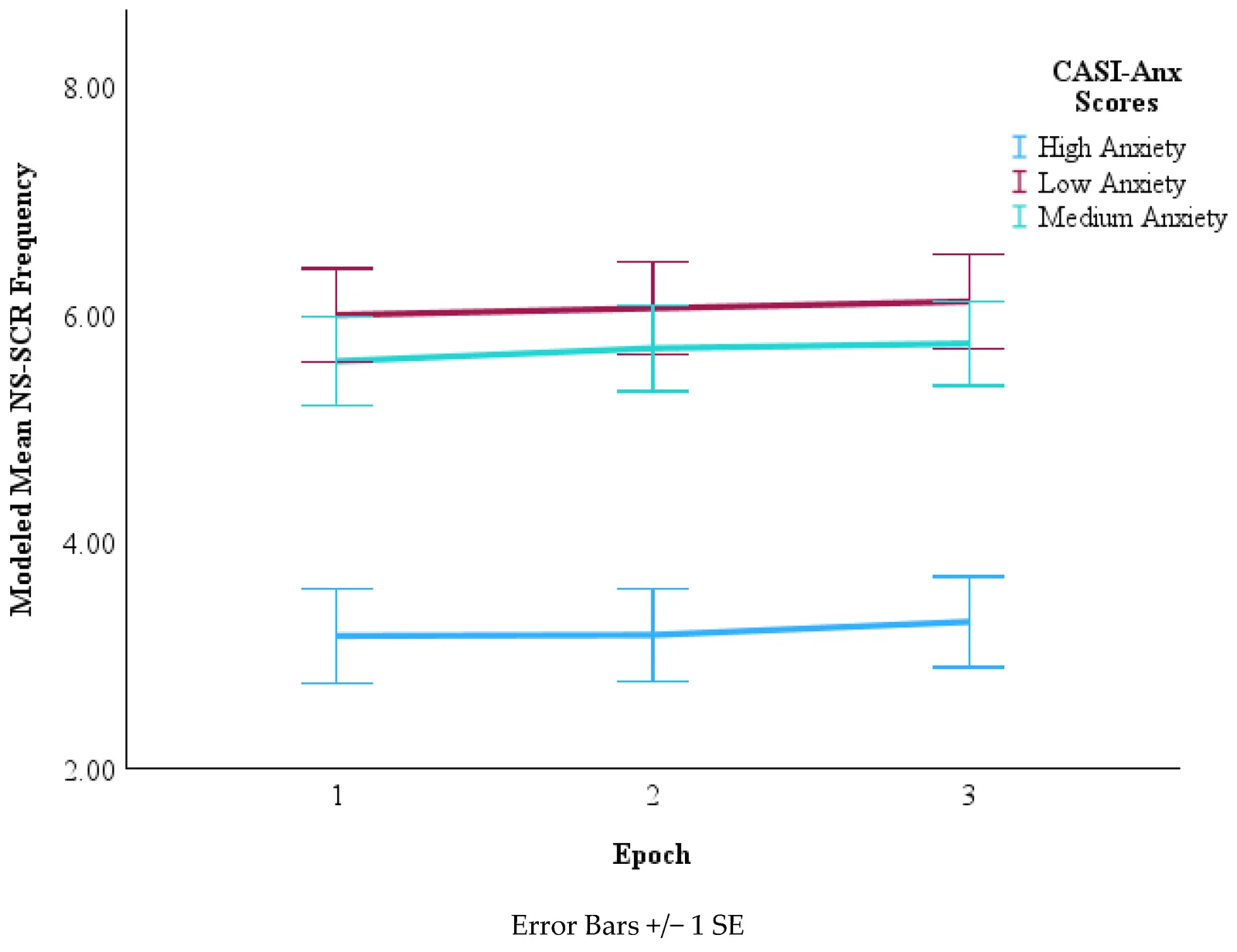
Graphical representation of changes in modeled frequency of non-specific skin conductance responses (NS-SCRs) by anxiety scores (CASI-Anx) across the three epochs.

Although there were no significant time × anxiety interactions within the linear growth model, higher CASI-Anx scores significantly predicted lower NS-SCR frequency (*b* = −0.07, *SE* = 0.03, *t*(121.68) = −2.07, *p* = 0.04), as shown in Figure 2. Additionally, though the curvilinear main effect of anxiety did not reach statistical significance within the unadjusted quadratic growth model (*b* = 0.10, *b*^2^ = −0.004, *p* = 0.12), controlling for individual differences in IQ via WASI-4 scores revealed a significant quadratic main effect of anxiety (*b*^2^ = −0.005, *SE* = 0.002, *t*(123.47) = −2.16, *p* = 0.03), indicating a stable, non-linear relationship between anxiety and baseline NS-SCR frequency, as shown in Table 6.

No significant main or interaction effects were found between phasic activity (NS-SCR frequency) and sensory over-responsiveness (*b* = −0.04, *p* = 0.16), autism traits (*b* = 0.12, *p* = 0.53), or expressive language (*b* = −0.23, *p* = 0.06), as shown in Table 7. The inclusion of CFSS-DS and WASI-4 scores as covariates did not alter the pattern of results or meaningfully improve model fit, which were all pseudo *R*^2^ ≤ 0.06, *p* ≥ 0.09.

## 4. Discussion

The current study examined the relationship between tonic (SCL) and phasic (NS-SCR) EDA arousal levels during a baseline rest period in a dental environment and autism-related participant characteristics (i.e., autism traits, anxiety, sensory responsivity, expressive language) in autistic children. Theories of resting-state hyper- [45] and hypo-arousal [36] have been proposed, both positing atypical arousal (i.e., a chronically under- or overreactive autonomic nervous system) as a unifying construct explaining differences in social communication skills and RRBs. Heightened SNS arousal is also associated with motivational systems and a readiness to act, engage, and learn—systems and processes frequently reported as atypical in autistic individuals [2,5,27]. Our findings contribute to the wider literature by characterizing relationships between resting-state EDA and participant characteristics within a large, clinically heterogeneous sample of autistic children, thus, contributing to our understanding of how underlying physiology intersects with environmental demands.

### 4.1. Tonic EDA Level and Phasic Activity

The results of the current study demonstrated a significant time-by-phasic (NS-SCR) EDA interaction predicting mean tonic (SCL) arousal. While the conditional main effect of time was negative, possibly indicating typical autonomic habituation and a linear drop in SCL for individuals with low spontaneous arousal activity [3,7,9], the positive interaction term indicates that higher baseline arousal was associated with a less pronounced drop (a flatter slope) over time. As expected, the observed relationship between SCL and NS-SCR frequency was consistent with the established physiological relationship between tonic and phasic measures of EDA [9,12,13,18,19,20] and supports the internal consistency of the EDA metrics examined in this study. Our findings replicate previously reported subgroups—(1) high SCL with slower habituation and (2) low SCL with faster habituation [12,19,20]—within a naturalistic dental environment.

### 4.2. Tonic EDA Level, Sensory Over-Responsiveness, Autism Traits, and Anxiety

In contrast with our hypothesis, participants with fewer autism traits (ADOS CSS scores) exhibited an ascending or sustained tonic (SCL) arousal trajectory across the baseline period, while participants with higher autism traits demonstrated a progressive, linear decline in SCL, which, based on the literature with non-autistic participants, suggests similar habituation patterns to non-autistic peers [11,18,21,22]. These results suggest that individual differences in autism traits may moderate SCL trajectories during a baseline rest period. Since these results were not substantially affected by the inclusion of the CFSS-DS (a measure of dental fear and anxiety), it is unlikely that participants with lower autism traits were reacting in anticipation of the upcoming dental exam alone. One possible explanation for these results is that children with fewer autism traits may experience more anticipatory anxiety [46]. Conversely, children with higher autism traits may experience elevated initial arousal upon entering the novel testing environment, followed by steady physiological habituation and a decline over the 3 min resting window [12]. Using two related indices of EDA variability (the standard deviation of the SCL and NS-SCR frequency), Fenning and colleagues [36] also generally support greater EDA within-task variability in participants with increased autism traits. In the context of these behavioral patterns, our longitudinal model examining the relationship between phasic and tonic EDA arousal supports Fenning and colleagues’ [36] findings and extends their results by further highlighting the possibility of a distinct high-tonic-EDA subgroup characterized by poor habituation during the 3 min baseline.

### 4.3. Phasic Activity, Sensory Over-Responsiveness, Autism Traits, Expressive Language, and Anxiety

While we hypothesized that increased anxiety would non-linearly predict more frequent NS-SCRs, our initial results indicated that the linear relationship between NS-SCRs and anxiety remained relatively stable over the three-minute period (hence the non-significant interaction effect), with a paradoxical, negative relationship between anxiety and NS-SCRs (i.e., as anxiety increased, NS-SCR frequency decreased). These initial linear results support the theory that children with high anxiety may be in a chronic state of high arousal, which may lead to a physiological ceiling effect whereby hyper-arousal may suppress spontaneous phasic activity (i.e., NS-SCRs), as suggested by Panju et al. [13]. This aligns with previous research indicating that autistic children with high anxiety may display dampened physiological responses [13,14,30].

However, a pivotal nuance emerged when we included IQ in our model; the quadratic effect of anxiety was not statistically significant until IQ was included as a covariate to control for the variation in participants’ cognitive functioning. After accounting for the effect of IQ, a suppressed, significant quadratic main effect of anxiety was revealed. This finding suggests that IQ may have acted as a suppressor variable, masking the underlying curvilinear relationship between anxiety and phasic activity.

This context provides crucial insight into the mixed findings that characterize the previous literature. Prior studies investigating autonomic profiles in autism, such as those by Panju et al. [13] and Parma et al. [14], have relied heavily on samples restricted to or characterized by high IQ ranges. Because cognitive variation can significantly confound physiological reactivity, their models did not have to contend with the masking effects present in more phenotypically heterogeneous samples [2]. For instance, previous research validating the CASI-Anx [41] in autistic children found that children with an IQ of 70 or greater showed significantly more caregiver-reported anxiety symptoms than those with an IQ less than 70, while minimally verbal children with IQs below 70 demonstrated the lowest anxiety symptoms [46]. Ultimately, these results support research showing that heightened chronic anxiety is associated with an autonomic ceiling or a restriction of phasic flexibility; this curvilinear physiological manifestation may be intertwined with—and possibly obscured by—individual differences in cognitive functioning.

### 4.4. Null Results

Our results did not support the hypothesized positive linear relationship between expressive language and phasic activity, nor sensory over-responsiveness with either tonic EDA level or phasic activity. The lack of association of EDA with either sensory over-responsiveness or expressive language aligns with recent findings suggesting that EDA captures a generalized state of sympathetic arousal rather than specific emotional or behavioral patterns [47]. Several methodological factors may explain these null findings.

#### 4.4.1. Methodological Considerations: Participant Characteristics

A primary methodological explanation for the null associations between EDA arousal and sensory over-responsiveness and expressive language may be the phenotypic heterogeneity of our sample compared with previous cohorts. Unlike the current study, most investigations reporting significant associations between SCL and participant characteristics—including sensory over-responsiveness and expressive language—excluded individuals with disruptive behaviors and significant intellectual delays [2,3,13,14,26,48]. This exclusion criterion in the existing literature may have inadvertently selected autistic participants with higher expressive language and lower sensory over-responsiveness [2,26,30]. Because previous studies showing significant associations between arousal and features like expressive language [23,24] and sensory responsiveness [12] relied on cohorts characterized by substantially higher mean IQs, our inclusion of a broader, more heterogeneous cognitive range may have reduced the ability to detect these associations, thereby contributing to the observed null results.

#### 4.4.2. Methodological Considerations: Measuring Sensory Responsivity Behaviors

Previous research on sensory responsivity and EDA has yielded mixed results across various modalities, including caregiver reports, sensory/self-stimulatory activities, and specific sensory stimuli [11,12,17,22]. Among studies linking EDA to caregiver-reported sensory responsivity, the results are inconsistent. For example, Chang et al. [17] found significant correlations with resting-state arousal, whereas, like the current study, McCormick et al. [22] and Schoen et al. [11] found no association between sensory responsivity and either tonic EDA level or phasic activity.

#### 4.4.3. Methodological Considerations: Procedural Differences, Autism Traits, and Expressive Language

Differences between the current study results and previous research may also relate to study procedures, such as measuring EDA during a baseline rest period rather than measuring sympathetic responses to naturalistic [10,23] and experimental [24] social stimuli. Autism traits [26] and arousal differences related to expressive language [23,24] may be context-dependent and more likely to be elicited in social interaction conditions. In contrast, the current study used data collected during baseline period with minimal social interaction immediately prior to a dental procedure. Thus, the socio communicative nature of tasks in previous studies examining arousal-related autism traits and expressive language associations may engage distinct SNS activation-related mechanisms that highlight social communication-associated phasic EDA activity.

#### 4.4.4. Methodological Considerations: Environmental Differences

Unlike previous studies examining resting-state EDA, the current study used data from a potentially stressful dental office environment immediately preceding care. Under stressful conditions, SNS activation stimulates eccrine sweat glands, which directly impact the skin’s electrical properties [9]. Although heterogeneity in SCL and NS-SCRs was observed in the current sample, and caregiver-reported specific dental fear and anxiety did not appear to impact the results of the current study based on analyses using dental fear and anxiety as a covariate, we do not have data on participants’ SCL and NS-SCRs outside of the dental environment. It is possible that the acute stress of the dental environment may have had an equalizing or masking effect across participants, reducing or negating the impact of behavioral or symptom-related differences observed in EDA results from studies conducted under less stressful conditions. The variance in the tonic and phasic EDA data may be better explained by individual reactions to the dental environment than by differences in ASD-related behavioral characteristics observed in other resting-state EDA results [17].

### 4.5. Limitations

The current study has several limitations that restrict the generalizability of our results. A primary limitation is our reliance on a single method of physiological measurement. Reliance on a single index of autonomic activity (e.g., EDA) may not adequately capture the complexities of autonomic arousal. As underscored by a recent systematic review [49] of SNS modeling, data relying on a single physiological modality are inherently vulnerable to individual baseline variations, task-specific interference, and environmental noise. Because EDA serves as a sensitive anchor for generalized physiological arousal rather than emotional or psychological valence, interpreting autonomic drive in isolation risks misclassifying diverse states of internal tension or engagement. To mitigate these limitations, future research should also integrate multiple psychophysiological markers (e.g., EDA, heart rate variability, pupillometry), behavioral observations (e.g., tactile defensiveness, attention-orienting), and caregiver reports to gain a holistic view of the child’s internal state and the form and function of their external behaviors.

While the present study utilized multi-level growth modeling to assess piecewise, epoch-to-epoch shifts across three one-minute baseline periods, future research could expand upon these findings by incorporating continuous signal-processing techniques. Specifically, time-frequency spectral analysis of EDA offers a framework for deriving continuous, time-varying sympathetic indices (e.g., spectral power in specific EDA frequency bands) across dynamic task conditions [6,50]. Combining continuous spectral analysis with multi-level modeling of discrete experimental phases may provide a more comprehensive characterization of both instantaneous autonomic reactivity and broader trajectory shifts in autistic children.

Another limitation was the use of secondary data collected at a dental office during a baseline period. Given the generally stressful nature of dental visits in this population [51,52,53], the baseline resting-state EDA data may have been influenced by participants’ learned stress response and anticipatory arousal as they waited for the dental procedure to begin. Our autistic sample may have already been in a state of hyper-arousal; however, without being able to compare their baseline in the dental environment to baseline EDA within a relaxing or neutral environment, this is only a possibility. As noted by Levinson and Hajcak [54], across-group associations and differences in autonomic arousal during baseline/rest conditions can be challenging to interpret, as these differences may reflect individual trait-like differences in autonomic arousal. Future research should also include resting-state data from a non-autistic control group and in a neutral or relaxing comparison condition.

Finally, as one of only a few studies examining the association between EDA arousal and autism-related participant characteristics, we took a relatively exploratory approach to data analysis. Because this approach, multiple comparisons were conducted. Future studies should use more restrictive hypothesis testing or use a single model to assess each predictor variable’s contribution to the variance explained in the outcome variable.

## 5. Conclusions

We utilized conditional growth modeling to assess the within-participant temporal relationship between tonic and phasic EDA during a 3 min baseline period, as well as the relationships between these EDA parameters and sensory over-responsiveness, autism traits, expressive language, and anxiety. In general, our results suggest that autism traits and anxiety predicted changes in EDA. Specifically, we observed a significant time by autism trait interaction for SCL and a significant linear and quadratic relationship between anxiety and NS-SCRs. Overall, our results support previous evidence of within-autism-population arousal patterns related to autism traits [12,19,21,26], anxiety [13,14], and tonic-to-phasic covariance patterns [28].

Beyond capturing baseline EDA arousal patterns, our results have practical and theoretical significance for understanding autistic heterogeneity prior to likely stressful clinical and community events, such as dental visits. Although the relationships between resting-state electrodermal metrics and participant characteristics (i.e., autism traits, anxiety) observed here are modest and exploratory, warranting cautious interpretation, the presence of temporal shifts in tonic EDA level (SCL) and phasic activity (NS-SCR) indicates that baseline sudomotor activity is neither static nor uniform across autistic children. Characterizing these dynamic variations in pre-treatment arousal profiles offers preliminary insight into why children may exhibit differing degrees of physiological vulnerability or distress before a medical procedure even begins. Rather than serving as standalone diagnostic predictors, these person- and phase-specific arousal parameters lay a foundation for understanding how underlying physiological state intersects with environmental demands. Clinically, recognizing this physiological variability underscores the importance of developing flexible, tailored supports (e.g., individualized sensory accommodations, modified waiting environment protocols, pre-procedure regulation strategies) that adapt to a child’s real-time physiological readiness rather than assuming uniform baseline functioning.

Differences and inconsistencies in the methods and measures used across the previous literature continue to limit comparison of EDA studies involving autistic children. Future research should replicate prior studies and compare results across multiple tonic and phasic EDA measures to help validate and establish consistent definitions, calculations, and procedures for EDA-related variables [28]. Additionally, further consideration should be given to addressing EDA arousal heterogeneity in autism through subtyping studies (e.g., latent profile analysis), which could better disentangle the possible associations between EDA measures and autism-related participant characteristics and help explain sympathetic arousal-related heterogeneity within the autism phenotype [28]. Despite the null associations found in this context, the continued use of EDA and other physiological measures is supported by their ability to provide objective physiological data that complement caregiver reports and behavioral observation [55].

## Figures and Tables

**Table 1:** Participant demographics.

Child	*n* (%)
Age	
mean years (SD)	9.28 (2.02)
Sex	
Male	113 (83.70)
Female	22 (16.30)
Race	
White/Caucasian	105 (77.78)
Black/African American	12 (8.89)
Asian	11 (8.15)
Unknown	6 (4.44)
Pacific Islander	1 (0.74)
Ethnicity	
Hispanic/Latino	95 (70.37)
Non-Hispanic/Latino	40 (29.63)
Co-occurring Diagnoses	
ADHD ^1^	26 (19.26)
Anxiety	6 (4.44)
Mother’s Education	
<High school	19 (14.07)
High school	20 (14.81)
Some college	54 (40.00)
College degree or more	42 (31.11)

Note. The descriptive statistics collected from the first consent visit represent the 135 participants with usable baseline EDA from the regular dental environment. *n* (%), unless marked as mean (SD). ^1^ Attention deficit hyperactivity disorder (ADHD).

**Table 2:** Child electrodermal activity (EDA) and behavioral summary scores and measures.

Scores and Measures	Mean (SD)
Mean SCL ^1^	7.43 (4.80)
NS-SCR ^2^ frequency	5.57 (3.39)
SensOR Inventory ^3^ total	28.15 (13.76)
SensOR Inventory, range	1–66
SensOR < 14, *n* (%)	21 (15.56%)
SensOR ≥ 14, *n* (%)	114 (84.44%)
ADOS-2 ^4^ calibrated severity scores ADOS-2 severity scores, range	6.30 (1.80) 3–10
Low-severity autism (3–4), *n* (%)	29 (21.48%)
Moderate-severity autism (5–7), *n* (%)	78 (57.78%)
High-severity autism (8–10), *n* (%)	28 (20.74%)
VABS-II ^5^	
Expressive language	8.65 (2.87)
Expressive language scores, range	3–19
Adequate > 12, *n* (%)	8 (5.97%)
Moderately low 10–12, *n* (%)	43 (32.09%)
Low ≤ 12, *n* (%)	83 (61.94%
CASI-Anx ^6^ total	21.15 (10.93)
CASI-Anx scores, range	0–50
WASI-II FSIQ4 ^7^	74.21 (24.14)
FSIQ4, range	40–126
<70, *n* (%)	62 (45.93%)
≥70, *n* (%)	73 (54.07%)
CFSS-DS ^8^ total	47.57 (13.78%)
CFSS-DS scores, range	16–75
CFSS-DS < 32, *n* (%)	23 (17.04%)
CFSS-DS ≥ 32, *n* (%)	112 (82.96%)

Note. The descriptive statistics represent the 135 participants with usable baseline regular dental environment electrodermal activity (EDA) data. Mean and standard deviation (SD), unless marked as range or number and percent. ^1^ Skin conductance level (SCL) and ^2^ non-specific skin conductance responses (NS-SCRs) are reported as non-normalized data. ^3^ Sensory Over-Responsivity (SensOR) Inventory (based on Schoen et al. [39] scores ≥ 14 indicates clinically relevant sensory over-responsiveness); ^4^ Autism Diagnostic Observation Scale-2 (ADOS-2); ^5^ Vineland Adaptive Behavior Scales-II (VABS-II) Expressive Communication (standardized scaled scores with a mean of 15 and standard deviation of 3); ^6^ CASI-4 Autism Anxiety Scale: Child and Adolescent Symptom Inventory-4, Autism Spectrum Disorder Anxiety Scale (CASI-Anx); ^7^ Wechsler Abbreviated Scale of Intelligence Full Scale Intelligence Quotient (WASI-II FSIQ4; scores less than 70 are indicative of intellectual disability); ^8^ Children’s Fear Survey Schedule-Dental Subscale (CFSS-DS; scores ≥ 32 are indicative of borderline to clinical dental fear).

**Table 3:** Summary of the linear conditional growth model of non-specific skin conductance responses (NS-SCRs) predicting the mean skin conductance level (SCL).

Parameter	Estimate (*b*)	*SE*	*df*
Fixed Effects			
Intercept	6.38	0.39	170.81
Time	−0.005 *	0.002	152.49
NS-SCR	0.11 **	0.03	233.76
Time X NS-SCR	0.001 **	0.00	167.06
Random Effects	Variance	*SE*	
Intercept	16.29	2.14	
Time Slope	0.00	0.00	
Intercept–Slope Covariance	0.004	0.01	
Residual	0.64	0.08	

* *p* < 0.05, ** *p* < 0.01.

**Table 4:** Summary of linear conditional growth models predicting mean skin conductance level (SCL) and sensory over-responsiveness, autistic traits, and expressive language.

	Sensory Over-Responsiveness		Autism Traits		Expressive Language	
Parameter	*b* (*SE*)	*df*	*b* (*SE*)	*df*	*b* (*SE*)	*df*
Fixed Effects						
Intercept	7.41 (0.92)	133.09	8.55 (1.43)	132.78	7.02 (1.30)	131.90
Time	0.00 (0.00)	130.65	0.01 (0.004) *	130.53	0.00 (0.00)	129.88
Main Effect	−0.01 (0.03)	132.94	−0.21 (0.22)	132.88	0.03 (0.14)	131.77
Time × Predictor	0.00 (0.00)	130.86	−0.001 (0.001) *	130.42	0.00 (0.00)	129.64
Random Effects	Variance (*SE*)		Variance (*SE*)		Variance (*SE*)	
Intercept	21.47 (2.73)		21.32 (2.71)		21.51 (2.75)	
Time Slope	0.00 (0.00)		0.00 (0.00)		0.00 (0.00)	
Intercept–Slope Covariance	0.01 (0.01)		0.01 (0.01)		0.01 (0.01)	
Residual	0.81 (0.10)		0.81 (0.10)		0.82 (0.10)	

* *p* < 0.05.

**Table 5:** Summary of linear conditional growth models predicting mean skin conductance level (SCL) by anxiety.

	Anxiety Linear Model		Anxiety Quadratic Model	
Parameter	*b* (*SE*)	*df*	*b* (*SE*)	*df*
Fixed Effects				
Intercept	7.22 (0.88)	125.58	5.26 (1.48)	124.52
Time	0.002 (0.003)	123.71	0.01 (0.004) *	122.57
Anxiety	0.00 (0.03)	125.51	−0.21 (0.22)	124.59
Anxiety^2^^†^			−0.004 (0.003)	124.68
Time × Anxiety	0.00 (0.00)	123.52	0.001 (0.00)	122.71
Time × Anxiety^2^^†^			0.00 (0.00)	122.74
Random Effects	Variance (*SE*)		Variance (*SE*)	
Intercept	21.63 (2.82)		21.33 (2.80)	
Time Slope	0.00 (0.00)		0.00 (0.00)	
Intercept–Slope Covariance	0.01 (0.01)		0.01 (0.01)	
Residual	0.77 (0.10)		0.77 (0.10)	

* *p* < 0.05; ^†^ 2 represents the quadratic regression model term.

**Table 6:** Summary of linear conditional growth models predicting frequency of NS-SCRs by anxiety.

	Anxiety Linear Model		Anxiety Quadratic Model		Anxiety Quadratic Model with IQ	
Parameter	*b* (*SE*)	*df*	*b* (*SE*)	*df*	*b* (*SE*)	*df*
Fixed Effects						
Intercept	6.78 (0.73)	121.53	5.24 (1.23)	120.99	7.30 (1.37)	132.03
Time	0.00 (0.01)	115.65	0.001 (0.01)	116.25	0.001 (0.01)	116.51
Anxiety	−0.07 (0.03) *	121.68	0.10 (0.11)	121.99	0.18 (0.11)	123.49
Anxiety^2^^†^			−0.004 (0.002)	122.77	−0.005 (0.002) *	123.47
Covariate (IQ)					−0.04 (0.01) **	121.31
Time × Anxiety	0.00 (0.00)	117.45	0.00 (0.001)	116.88	0.00 (0.001)	117.13
Time × Anxiety^2^^†^			0.00 (0.00)	116.77	0.00 (0.00)	117.02
Random Effects	Variance (*SE*)		Variance (*SE*)		Variance (*SE*)	
Intercept	10.19 (2.08)		10,036 (2.06)		8.92 (1.94)	
Time Slope	0.00 (0.00)		0.00 (0.00)		0.00 (0.00)	
Intercept–Slope Covariance	−0.01 (0.01)		−0.01 (0.01)		−0.01 (0.01)	
Residual	5.48 (0.72)		5.48 (0.72)		5.49 (0.71)	

* *p* < 0.05, ** *p* < 0.01, ^†^ 2 represents the quadratic regression model term.

**Table 7:** Summary of linear conditional growth models predicting frequency of NS-SCRs by sensory over-responsiveness, autistic traits, and expressive language.

	Sensory Over-Responsiveness		Autism Traits		Expressive Language	
Parameter	*b* (*SE*)	*df*	*b* (*SE*)	*df*	*b* (*SE*)	*df*
Fixed Effects						
Intercept	6.42 (0.79)	129.79	4.67 (1.25)	129.32	7.36 (1.10)	129.68
Time	0.00 (0.01)	114.93	0.01 (0.01)	127.71	−0.003 (0.01)	118.58
Main Effect	−0.04 (0.03)	129.40	0.12 (0.19)	129.96	−0.23 (0.12)	129.98
Time × Predictor	0.00 (0.00)	117.62	−0.001 (0.001)	125.77	0.001 (0.001)	120.03
Random Effects	Variance (*SE*)		Variance (*SE*)		Variance (*SE*)	
Intercept	10.75 (2.07)		10.96 (2.09)		10.63 (2.07)	
Time Slope	0.00 (0.00)		0.00 (0.00)		0.00 (0.00)	
Intercept–Slope Covariance	−0.01 (0.01)		−0.01 (0.01)		−0.01 (0.01)	
Residual	5.36 (0.68)		5.37 (0.68)		5.37 (0.69)	

## Data Availability

Deidentified data and a corresponding data dictionary will be made available to interested researchers following completion of publications and research presentations addressing the primary and major secondary analyses of this study. Access will be granted to researchers following review and approval of a brief proposal describing the intended use of the data. Interested parties should contact the last author to request access.

## Supplementary Materials

The following supporting information can be downloaded at: https://www.mdpi.com/article/10.3390/s26196172/s1, Table S1: Description of SADE-2 study behavioral assessments. References [39,40,41,42,56] are cited in the Supplementary Materials.

## Abbreviations

The following abbreviations are used in this manuscript:

EDA	Electrodermal activity
RRBs	Restricted and repetitive behaviors
SNS	Sympathetic nervous system
SCL	Skin conductance level
SCR	Skin conductance response
NS-SCR	Non-specific skin conductance response
ADOS-2	Autism Diagnostic Observation Scale
CSS	Calibrated Severity Scores
ANS	Autonomic nervous system
SADE	Sensory-adapted dental environment
ADHD	Attention deficit hyperactivity disorder
SensOR	Sensory Over-Responsivity Inventory
VABS-II	Vineland Adaptive Behavior Scales, 2nd ed.
CASI-Anx	Child and Adolescent Symptom Inventory-4: Autism Anxiety Scale
WASI-II	Wechsler Abbreviated Scale of Intelligence, 2nd ed.
CFSS-DS	Children’s Fear Survey Schedule–Dental Subscale
μS	Microseimens
