# Supplementary material for: Electrodermal Activity as a Metric of Arousal in Autistic Children Prior to Dental Treatment
Source: Sensors (Basel). 2026 Sep 29;26(19):6172. doi: 10.3390/s26196172 (PMC13667661; doi:10.3390/s26196172)
Supplement: Supplementary file 1 [file sensors-26-06172-s001.zip › sensors-4344607-supplementary.pdf]

Supplemental Materials

Table S1. Description of SADE-2 study behavioral assessments

Assessment	Scoring
ADOS-2	Total scores range from 15-60. Calibrated Severity Scores were computed using an algorithm that entered selected items[1]. Scores range from 1-10, with higher scores indicating more frequent or more prominent autism traits[2]. Participants with scores of 1-2 were excluded.
SensOR Inventory	76-item caregiver-report scale with six domains (tactile, auditory, visual, olfactory, food texture, and movement-proprioceptive). Items were scored *yes(1)* or *no(0)*. Total summed scores ranged from 0-76, with higher scores indicating greater sensory over-responsivity[3].
Vineland Adaptive Behavior Scales, 2nd Ed (Vineland-2): Communication Domain, Expressive Language Subscale	Items are scored on a 0-2 point scale based on the independent performance of various adaptive living tasks (*0-never, 1-sometimes/partially, 2-usually performed*)[4]. Standardized V-Scale scores were used for analysis. Raw scores from the VABS-II Expressive Language subscale were converted to standardized V-Scale scores (*m*=15, *SD*=3).
Child and Adolescent Symptom Inventory-4 (CASI-4): Autism Anxiety Scale (CASI-Anx)	20-item caregiver-report measure scored on a 4-point scale from *0-never* to *3-very often*. Range of 0-60, with higher total scores indicating greater generalized anxiety[5]. Individual item scores of 2 or 3 are considered clinically relevant. Total scores were used for this study.

References

1. Gotham, K.; Pickles, A.; Lord, C. Standardizing ADOS scores for a measure of severity in autism spectrum disorders. J Autism Dev Disord 2009, 39, 693–705. https://doi.org/10.1007/s10803-008-0674-3.
2. Lord, C.; Luyster, R.J.; Gotham, K.; Guthrie, W. Autism Diagnostic Observation Schedule (ADOS-2) Manual, 2nd ed.; Western Psychological Services: Torrance, CA, USA, 2012.
3. Schoen, S.A.; Miller, L.J.; Green, K.E. Pilot study of the sensory over-responsivity scales: assessment and inventory. Am J Occup Ther 2008, 62, 393–406. https://doi.org/10.5014/ajot.62.4.393.
4. Sparrow, S.S.; Cicchetti, D.V.; Balla, D.A. Vineland Adaptive Behavior Scales, 2nd ed.; American Guidance Service: Circle Pines, MN, USA, 2005. https://doi.org/10.1037/t15164-000.
5. Sukhodolsky, D.G.; Scahill, L.; Gadow, K.D.; Arnold, L.E.; Aman, M.G.; McDougle, C.J.; McCracken, J.T.; Tierney, E.; White, S.W.; Lecavalier, L.; Vitiello, B. Parent-rated anxiety symptoms in children with pervasive developmental disorders: frequency and association with core autism symptoms and cognitive functioning. J Abnorm Child Psychol 2008, 36, 117–128. https://doi.org/10.1007/s10802-007-9165-9.
